# Effect of Shear History on Rheology of Time-Dependent Colloidal Silica Gels

**DOI:** 10.3390/gels3040045

**Published:** 2017-11-20

**Authors:** Paulo H. S. Santos, Marcelo A. Carignano, Osvaldo Campanella

**Affiliations:** 1 Department of Agricultural and Biological Engineering, Purdue University, 225 South University Street, West Lafayette, IN 47907, USA; 2 Qatar Environment and Energy Research Institute, Hamad bin Khalifa University, P.O. Box 34110 Doha, Qatar; mcarignano@hbku.edu.qa

**Keywords:** gel, silica, rheology, thixotropy, microstructure, viscosity, JP-8, fuel

## Abstract

This paper presents a rheological study describing the effects of shear on the flow curves of colloidal gels prepared with different concentrations of fumed silica (4%, 5%, 6%, and 7%) and a hydrophobic solvent (Hydrocarbon fuel, JP-8). Viscosity measurements as a function of time were carried out at different shear rates (10, 50, 100, 500, and 1000 s^−1^), and based on this data, a new structural kinetics model was used to describe the system. Previous work has based the analysis of time dependent fluids on the viscosity of the intact material, i.e., before it is sheared, which is a condition very difficult to achieve when weak gels are tested. The simple action of loading the gel in the rheometer affects its structure and rheology, and the reproducibility of the measurements is thus seriously compromised. Changes in viscosity and viscoelastic properties of the sheared material are indicative of microstructural changes in the gel that need to be accounted for. Therefore, a more realistic method is presented in this work. In addition, microscopical images (Cryo-SEM) were obtained to show how the structure of the gel is affected upon application of shear.

## 1. Introduction

For a wide range of complex fluids such as gels, their flow properties are not a function of shear rate alone [1]. Depending on the type of gel, temperature, and concentration, among other conditions, gels can exhibit either solid or liquid viscoelastic behavior, which is commonly found in materials, notably colloidal gels, that can form networks due to the presence of non-covalent bonds. A challenge during the testing of gels is that flow and deformation can induce reversible and irreversible structural changes, making them time or deformation dependent fluids [2].

Changes in the viscosity or viscoelastic properties of colloidal gels as a function of time, flow, and deformation are relevant in several industrial processes and product formulations. For example, the texture and sensorial characteristics of some food products depend on the processing steps and on the shear history to which the product is subjected during manufacturing [3,4,5]. The change with time of the rheological properties of pharmaceutical formulations may influence the controlled release of loaded drugs, defining their therapeutic efficacy and bioavailability [6]. In colloid science, the decrease of viscosity of gels and suspensions of colloidal particles when shear is applied make them key components in mining, ceramic, and coating industries [7,8,9,10,11]. Moreover, time dependent colloidal gels are an emerging alternative in biotechnology for cell culture [12] and in rocket propulsion systems [13,14,15].

The reversible time dependent rheological phenomenon of fluids in which the viscosity of these materials decreases with time and/or deformation is referred to as Thixotropy, whereas the opposite phenomenon is known as Rheopexy. For Thixotropic fluids, their viscosity decreases with time when a constant shear is applied; however, their viscosity may be fully or partially recovered when the shear is ceased [16]. The microstructural changes and mechanisms that govern this transition are quite complex and not very well understood, especially in colloidal systems [12,13,14,15,16,17,18,19]. Colloidal suspensions can aggregate to form a percolated network or gel when the system is destabilized [20,21,22,23]. The resulting network formed by weak, mostly non-covalent, attractive forces gives the system solid-like viscoelastic properties. Most often, the thixotropic behavior of these fluids is related to changes in their network which is “broken” by mechanical stresses and the rheology of the gel transitions to that of a viscoelastic liquid or a paste. The reduction or absence of the applied shear often allows the system to re-build the particulate network and consequently to recover its initial conformation and viscosity.

Irreversible or partial microstructure changes may also occur in colloidal systems [2]. Fumed silica, which is applied in many industrial activities, can form distinctive 3D gel networks at relatively low concentrations. The surface of these colloidal particles plays an important role in the formation of gels: the presence of hydrophilic groups makes the particles capable of building hydrogen bonds between themselves to create a percolated network [24]. Because of their hydrophilic properties, a strong particle-particle interaction is observed in the presence of hydrophobic solvents, resulting in the formation of a gel network at low concentrations of particles. In contrast, a higher particle concentration is needed for gel formation if the solvent has hydrophilic characteristics. In this case, there is competition between particle-particle and particle-solvent interactions. In addition to the nature of the solvent, other parameters may affect the rheological properties of the system, including the concentration of particles, degree of dispersion, and temperature. Previous reports showed that these may also affect the rheological time dependence of colloidal gels [13].

Although time dependent fluids have been an object of study for many years, there are still few extensive accurate data available in the literature, in particular, for gels with a very fragile structure that are unquestionably disturbed when loading them in the rheological testing instrument. Thus, the characterization and a suitable rheological representation of these systems are notoriously challenging and experimental data in a wide range of conditions would be extremely helpful in solving open issues in thixotropic systems [25,26,27].

The objective of this work was to investigate and describe the macroscopic behavior of time dependent silica gels as a consequence of microstructural changes induced by shear. Viscosity data as a function of time was used to model irreversible flow property changes by incorporating a newly defined structural decay parameter that can be included in models used to describe time independent flow curves of fluids; for example, when studying the extended Herschel-Bulkley rheological model used in this work. In addition, qualitative data showing how the shear history affects viscoelastic parameters such as normal stresses measured during steady shear flow, as well as cryo-scanning electron micrographs (Cryo-SEM) of sheared materials, are presented.

Related work studying the rheology of these materials clearly shows that the shear history is a key parameter to be considered in the analysis because it strongly affects the properties of the gels, to the extent that it is recommended to pre-shear the material before testing [28]; this reinforces the importance of the studies presented in this manuscript.

## 2. Theory

Different approaches have been used to incorporate time dependence into rheological models. Basically, they can be divided into three classes: the continuum mechanics approach, and the use of structural kinetics and microstructural models. The first approach is not used in this work; instead, it focuses on a combined approach involving a structural kinetic model where the shear history effect and the material breakdown is incorporated by a microstructural model defined by the parameter *λ* that expresses the degree of structure of the material. Most models applied to time dependent fluids, such as the Tiu and Boger [29] model, also use a structural parameter, denoted by λ. The value of λ varies from a value of $\lambda_{0}$, indicating that the material is initially “intact” and its structure is fully developed at the initial time *t =* 0, to an equilibrium value ($\lambda_{e}$) that corresponds to a completely broken structure due to flow or deformation. In a more recent work, Denn and Bonn [30] proposed the description of the thixotropic behavior of complex fluids, such as gels, by a parameter that is a connectivity of the material structure with its flow properties.

Our preliminary work [13] has shown that for rheologically highly time dependent gels, such as those studied in the present study, it is extremely difficult, if not impossible, to obtain a flow curve that represents the rheology of the intact gel system. As the sample is highly affected by shear, the flow curve obtained describes a transient state of the fluid that depends on the measurement and the instrument loading conditions. As discussed, the simple action of loading the sample into the rheometer is enough to disturb its structure, which can seriously compromise the reproducibility of the measurements. To avoid that source of error, in the present study, the flow curve of the completely broken gel is used as the reference curve to define the structural parameter in the constitutive equation. In a way, the approach to assess the rheology of these gel materials is taking into consideration practical advises concerning pre-shearing the material before testing. Thus, samples are pre-sheared to eliminate time effects on the data and the flow curve of the broken system is measured and described by a more suitable rheological model. The extended Herschel-Bulkley model was used to describe the flow curve of the material expressed as shear stress τ as a function of the shear rate $\dot{\gamma }$ of the fully broken gel, i.e., when the structure of the gel is broken, and the gel behaves as a rheologically time independent material described by the following equation [13]:

(1) $$\tau =\tau_{0}+K{\dot{\gamma }}^{n}+\eta_{\infty }\dot{\gamma }$$

Therefore, to describe a gel with time dependent rheological properties, the structural parameter $\lambda \left(\dot{\gamma },t\right)$, which depends on the shear rate and time, is incorporated into the model as:

(2) $$\tau \left(\dot{\gamma },t\right)=\lambda \left(\dot{\gamma },t\right)\cdot \;\left[\tau_{0}+K{\dot{\gamma }}^{n}+\eta_{\infty }\dot{\gamma }\right]$$

where $\tau_{0}$ is the fluid yield stress, *K* is the consistency index, *n* is the power law index, and $\eta_{\infty }$ is the equilibrium viscosity, estimated as the viscosity of the gel when large shear rates are applied. In this work, the viscosity was determined by performing a measurement in a high shear rate capillary viscometer.

The structural parameter $\lambda \left(\dot{\gamma },t\right)$ characterizes the changes of the gel structure with shear rate and time, and it is assumed to follow a second order kinetic equation:

(3) $$\frac{d\lambda \left(\dot{\gamma },t\right)}{dt}=-c\left(\dot{\gamma }\right){\left[\lambda \left(\dot{\gamma },t\right)-\lambda_{e}\right]}^{2}$$

where $c\left(\dot{\gamma }\right)$ is a shear rate depending parameter, whereas $\lambda \left(\dot{\gamma },t\right)$ is a parameter that describes changes of the gel structure with time at a specific shear rate. It varies from an initial value *λ*_0_, representing the initial structure of the gel, to an asymptotic value $\lambda_{e}$, which will eventually be equal to 1 when the structure of the material is completely broken, i.e., when it can no longer be altered. However, in subsequent derivations, it will be left as $\lambda_{e}$ for the sake of generalization.

Equation (3) can be integrated from an initial time *t* = 0, when the structural parameter is $\lambda_{0}$, to a time *t* under which the structural parameter reaches the value λ(t) to yield:

(4) $$\lambda \left(\dot{\gamma },t\right)=\frac{\lambda_{0}+\lambda_{e}\cdot c\left(\dot{\gamma }\right)\cdot t\cdot \left(\lambda_{0}-\lambda_{e}\right)}{1+c\left(\dot{\gamma }\right)\cdot t\cdot \left(\lambda_{0}-\lambda_{e}\right)}$$

Thus, provided that the parameter $c\left(\dot{\gamma }\right)$ is known, the variation of the structural parameter $\lambda \left(\dot{\gamma },t\right)$ of the gel can be determined by Equation (4). Determination of the parameter $c\left(\dot{\gamma }\right)$ can be performed by measuring the viscosity of the gel system with a function of time at fixed shear rates.

From Equation (2) and the definition of an apparent viscosity, $\eta \left(\dot{\gamma },t\right)=\;\frac{\tau \left(\dot{\gamma },t\right)}{\dot{\gamma }}$, the following equation is obtained:

(5) $$\lambda \left(\dot{\gamma },t\right)=\frac{\eta \left(\dot{\gamma },t\right)\;\dot{\gamma }}{\left[\tau_{0}+K{\dot{\gamma }}^{n}+\eta_{\infty }\dot{\gamma }\right]}$$

As noted in Equation (5), the apparent viscosity of the gel $\eta \left(\dot{\gamma },t\right)$ is a function of the shear rate $\dot{\gamma }$ and time *t* and accounts for the changes in the structural parameter $\lambda \left(\dot{\gamma },t\right)$. By differentiating Equation (5) with respect to time and combining it with Equation (3), the following relation is obtained:

(6) $$\frac{d\eta \left(\dot{\gamma },t\right)}{dt}=\frac{c\left(\dot{\gamma }\right)\cdot \left[\tau_{0}+K{\dot{\gamma }}^{n}+\eta_{\infty }\right]\cdot {\left[\lambda \left(t\right)-\lambda_{e}\right]}^{2}}{\dot{\gamma }}$$

Equation (6) can be rearranged as:

(7) $$\frac{d\eta \left(\dot{\gamma },t\right)}{dt}=-a\left(\dot{\gamma }\right){\left[\eta \left(\dot{\gamma },t\right)-\eta_{e}\right]}^{2}$$

where:

(8) $$a\left(\dot{\gamma }\right)=\frac{c\left(\dot{\gamma }\right)\;\dot{\gamma }}{\left[\tau_{0}+K{\dot{\gamma }}^{n}+\eta_{\infty }\;\dot{\gamma }\right]}$$

and

(9) $$\eta \left(\dot{\gamma },t\right)=\lambda \left(\dot{\gamma },t\right)\left[\frac{\tau_{0}+K{\dot{\gamma }}^{n}+\eta_{\infty }}{\dot{\gamma }}\right]$$

**Figure 1:**
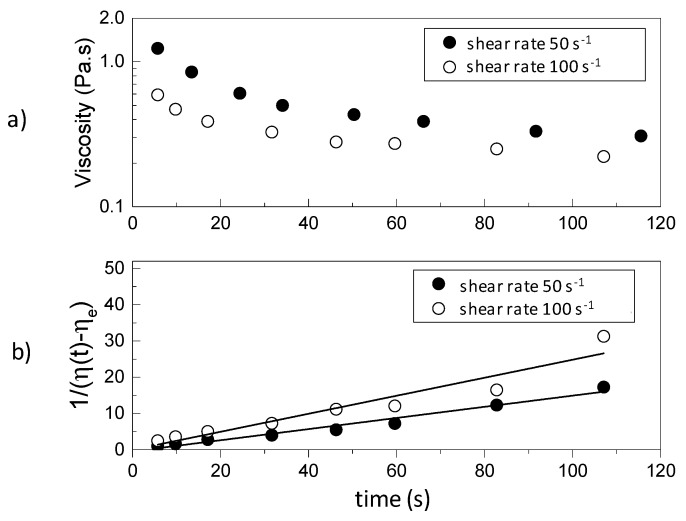
(**a**) Typical thixotropic behavior of a rheological time dependent gel after the application of constant shear rates of 50 1/s and 100 1/s; (**b**) schematic of the approach used to determine the parameters defining the structural parameter $\lambda \left(\dot{\gamma },t\right)$ from Equation (10). Data used are from a 5% silica gel.

A typical schematic thixotropic behavior of a rheological time dependent gel after the application of constant shear rates of 50 1/s and 100 1/s is depicted in Figure 1a. By integrating Equation (6) between *t* = 0 and an arbitrary time *t*, the following equation is obtained:

(10) $$\frac{1}{\eta \left(\dot{\gamma },t\right)-\eta_{e}}=\frac{1}{\eta_{0}-\eta_{e}}+a\left(\dot{\gamma }\right)t$$

A plot of $\frac{1}{\eta \left(\dot{\gamma },t\right)-\eta_{e}}$ versus *t*, at a constant shear rate, gives a straight line with slope $a\left(\dot{\gamma }\right)$, from which the value of $c\left(\dot{\gamma }\right)$ can be calculated from Equation (8), provided the rheological model of the sheared unstructured gel is known. Once the relevant parameters are known, Equation (4) can be used to estimate the variation of structural parameter $\lambda \left(\dot{\gamma },t\right)$ at different times and shear rates and thus predict the corresponding value of the viscosity by Equation (9).

## 3. Materials and Methods

### 3.1. Gel Preparation

Colloidal gels were prepared by adding fumed silica (CAB-O-SIL^®^, M-5, Cabot Corporation, Boston, MA, USA) with an average particle size of 0.2–0.3 µm and a surface area of 200 m^2^/g to a hydrophobic solvent (hydrocarbon fuel, kerosene grade JP-8, NATO code F-34, minimum density of 775.0–840 kg/m^3^ at 15 °C; minimum flash point of 37–38 °C; boiling point 157–300 °C; freezing point of −47 °C; maximum aromatics content of 25.0% *v*/*v* and maximum sulphur content of 0.30% mass). Samples were mixed using an acoustic mixer (Resodyn Resonant Acoustic^®^ LabRAM, Butte, MT, USA) to form the network. The acceleration of the mixer for the gel preparation was approximately 100 G for 60 s. The mixing conditions were based on the flow properties and stability of previously prepared gels [31]. The rheological experiments were conducted within 24–48 h after gels were made.

### 3.2. Microscopy

After the application of a controlled shear in a rheometer, the gel was immersed into liquid nitrogen and transferred to the Gatan Alto 2500 pre-chamber (cooled to −170 °C). The samples were directly transferred to the microscopy unit within a 30 min period for testing. Samples were imaged with an FEI Quanta 3D field emission scanning electron microscope (FEI company, Hillsboro, OR, USA) using the ET (Everhart-Thornley) detector operating at a 5 kV accelerating voltage, ~6 mm working distance, and 30 µm aperture. After fracturing the sample with a cooled scalpel to produce a free-break surface, the sample was not sublimated since it did not contain water. The samples were sputter coated for 90 s with platinum and then transferred to the microscope cryostage (−130 °C) for imaging. Time elapsed between the application of shear to the gels and the preparation of the micrographs (about 30 min) was sufficient to assume that the gel structure had not changed. This was confirmed by measuring the sample viscosity after 30 min resting and finding that it had not changed, and the same was true for the sample structure.

### 3.3. Rheological Characterization

Rheological characterization was carried out in triplicate using a rotational AR-G2 Rheometer with Smart SwapTM Geometry (TA Instruments, New Castle, DE, USA). A cone and plate geometry was used and all measurements were performed at a constant temperature of 25 °C. This geometry provided an almost uniform shear field. Slip is an artifact that is often unavoidable during rheological measurements; however, the samples were sticky and slippage did not appear to be a major problem with these samples. A parallel plate geometry could help to assess whether or not slippage was present in the measurements, but since this geometry does not provide an uniform shear field, the use of the cone and plate geometry was preferred to eliminate potential problems related to the interference of the yield stress in the measurements [30], which could be significant in this type of gel. A capillary rheometer (RH2000, Bohlin Instruments, Cirencester, UK) was used to obtain viscosities at a shear rate >1000 s^−1^ and the value of $\eta_{\infty }$.

## 4. Results and Discussion

**Figure 2:**
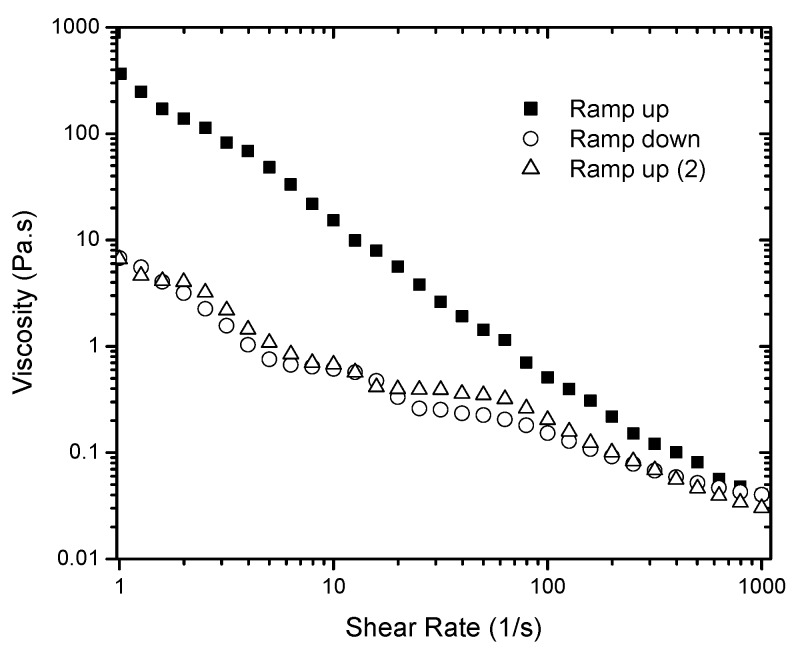
Viscosity measurements of 6% gel. Three runs were carried out using the same sample: shear rate was increased from 1 to 1000 s^−1^ (ramp up), varied from 1000 to 1 s^−1^ (ramp down), and increased from 1 to 1000 s^−1^ (ramp up 2).

Three flow curves were measured for each sample in a range of shear rates from 1 to 1000 s^−1^. Viscosity values were first obtained by applying an increasing shear rate ramp, followed by a decreasing shear rate ramp (1000 to 1 s^−1^), and finally by another increasing ramp (1 to 1000 s^−1^) test. Figure 2 presents the data obtained for a 6% gel. Typical time dependent materials exhibit different flow curves whenever a history of pre-shear exists, and the area of the hysteresis loop shows evidence of time dependence in the fluid. A significant difference is observed between the first ramp up and the ramp down measurements. Therefore, the shear forces applied to the sample during the first ramp up appear to be high enough to completely break down the gel network. No hysteresis loop was observed when the second ramp up and a third ramp down were compared. In addition, no viscosity recovery was observed. The gel structure is originally formed after the silica particles are highly dispersed in the solvent, which allows the formation of hydrogen bonds between the particles leading to the development of the percolated network. The shear applied may affect this network and consequently the gel rheology. Letting the system rest by itself did not recover its structure, at least within the time scale investigated (up to 30 min). This can be understood by considering that energy is needed for the initial formation of the gel (applied by the acoustic mixer) and the thermal motion of the particles was not enough to re-build the network.

**Figure 3:**
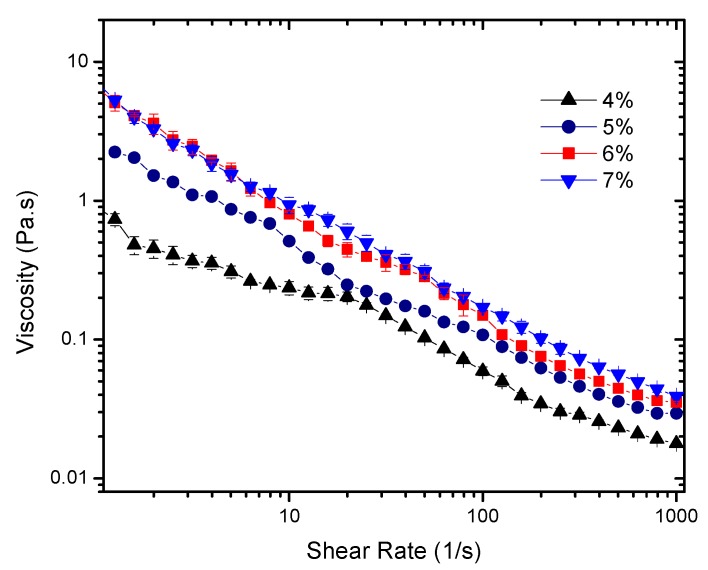
Viscosity (mean) as a function of shear rate for 4%, 5%, 6%, and 7% gels. These curves describe the flow behavior of the sheared and time independent fluid. The Extended Herschel-Bulkley model was applied to describe the flow characteristics of the gels. Measurements were carried out in triplicate. Error bars shown in the plot were calculated using a 95% confidence interval determined with the software OriginPro 2016 (OriginLab Corporation, Northampton, MA, USA).

The results indicate that the combined effect of time and shear induces irreversible changes for all concentrations. This allows the determination of the flow curves of the “broken” sheared gel (Figure 3) and the use of a time independent rheological model to describe the system. For that, the extended Herschel-Bulkley model $\tau =\tau_{0}+K{\dot{\gamma }}^{n}+\eta_{\infty }\dot{\gamma }$ was applied to describe their flow characteristics and to obtain the parameters reported in Table 1. Note that *η*_∞_ has been experimentally measured by a capillary rheometer at shear rates >10,000 s^−1^.

Few observations should be highlighted to show the strong time and shear dependence of these gels. First, significantly lower viscosity values were obtained for pre-sheared gels (up to 60 times lower). Second, the power law indexes for the pre-sheared gels were in the range 0 < *n* < 1, which characterizes them as shear thinning fluids. Inconsistent negative power flow indexes are no longer observed, as previously reported [13].

**Figure 4:**
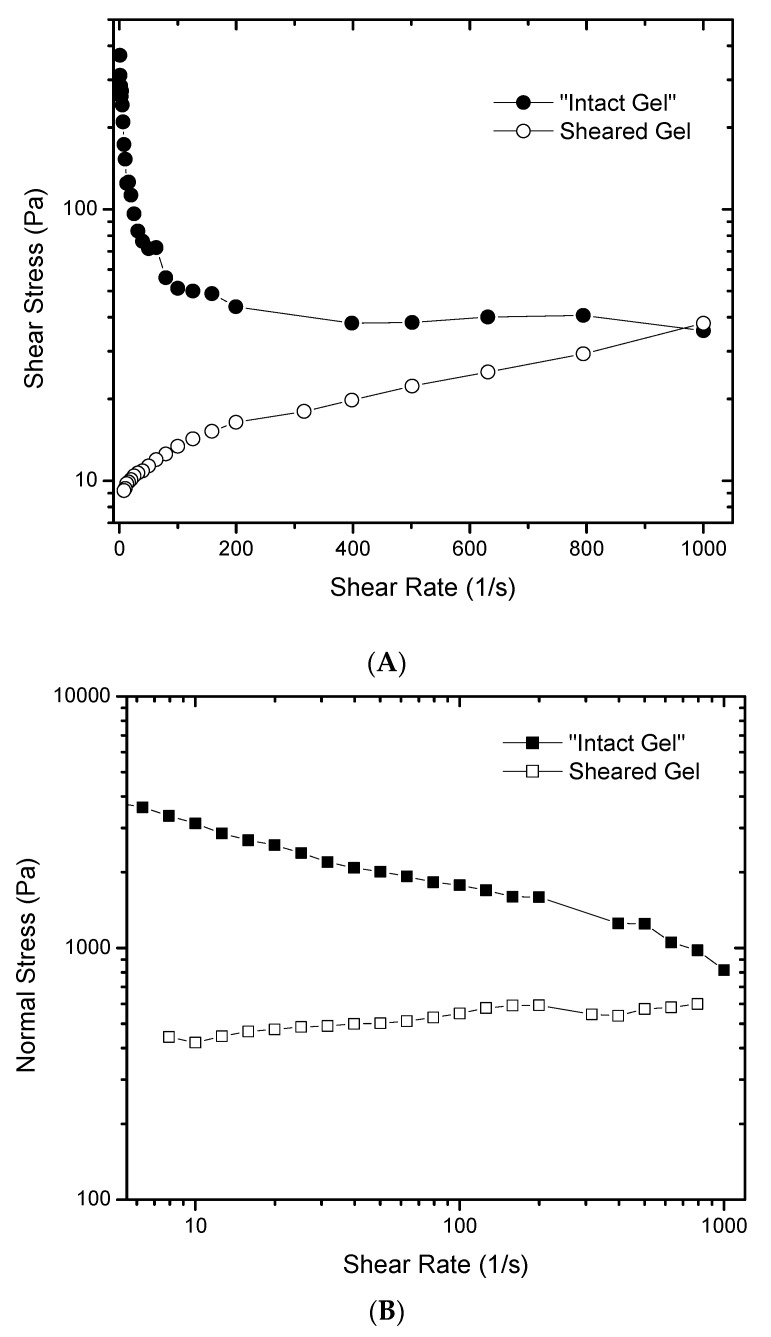
“Intact” and sheared gels: (**A**) shear stress versus shear rate of 6% gels. A typical curve of shear thinning fluid is only observed for the sheared gel (empty circle marks); (**B**) first normal stress versus shear rate of 6% gels. Filled squares show the decrease in normal stress of the “intact” gel at different shear rates. Empty squares show no variation of normal stress at different shear rates.

To further understand the rheological behavior of such gels, Figure 4 was created to show that the flow curve of the “intact” gel does not follow the typical behavior of a purely shear thinning fluid where shear stress increases with shear rate, albeit not in a linear manner (Figure 4A). In this case, when the shear rate is increased, the shear stress decreases, which would be an impossible rheological behavior for a material that does not undergo structural modifications upon shear. It also shows that the material is changing during the measurement due to the application of shear. Complementary data is provided in Figure 4B, showing that the first normal stress of the “intact” gel decreases when the shear rate goes up. The first normal stress is an indication of the elasticity of the material, generated by the gel network, that is being broken down by the effect of shear. After pre-shearing the gel, little dependence of the normal stress with shear is observed. Furthermore, the magnitude of the normal stress is smaller than the values measured for the “intact” gel. This is strong evidence that there are structural changes in the gel during deformation.

**Figure 5:**
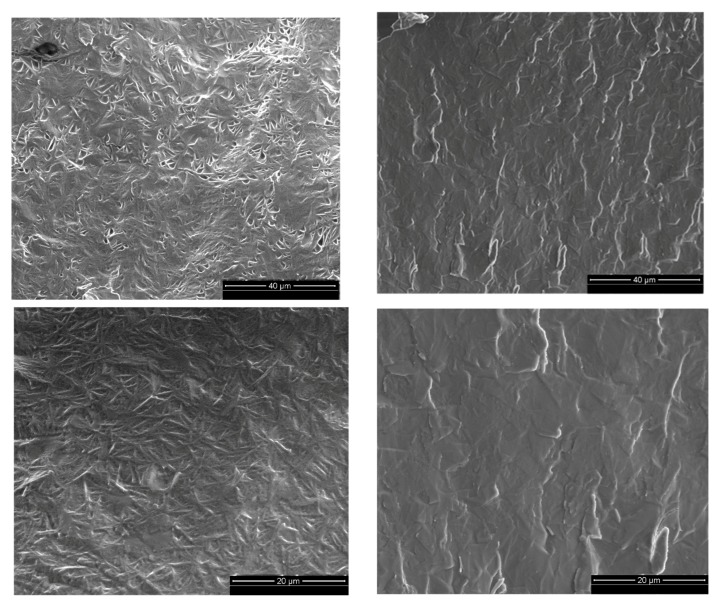
Cryo-SEM micrographs of 6% “intact” (**on the left**) and pre-sheared gels (**on the right**). Micrographs at different magnifications: 40 and 20 µm (from **top** to the **bottom**).

Sheared and “intact” gels were also analyzed by scanning electron microscopy). The micrographs illustrated in Figure 5 show different patterns for the two systems, especially at low magnifications. Both gels did have a primary structure of small particles closely packed together that were visible at higher magnifications. However, in the low magnification pictures, the unsheared sample seems to have a more organized structure with what appeared to be a cross-hatch pattern. After shear is applied, this texture is no longer present (right micrographs) and the sample had a more plate-like appearance. It is important to mention that the presence of a pattern (intact gel) or lack of a pattern (sheared gel) observed was consistent over the entire fractioned sample area. This difference in the texture of both gels at larger scales can be an additional indication of the changes in the organizational structure of the system caused by shear.

### Structural Kinetics Model

**Figure 6:**
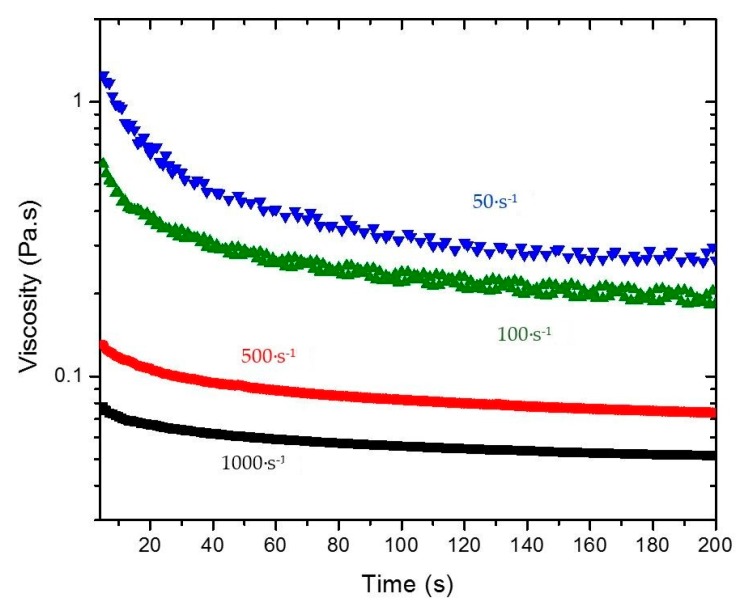
Viscosity of 4% “intact” gel as a function of time at different constant shear rates (50, 100, 500, and 1000 s^−1^).

By shearing the material at a given shear rate, viscosity values can be recorded as a function of time. In this way, time and shear are no longer coupled as they are in the ramp up and ramp down measurements shown in Figure 3. The shear history can then be incorporated in terms of a kinetic parameter that relates the viscosity with the structural changes in the material, expressed by Equation (4). Therefore, viscosity measurements as a function of time were performed at five different shear rates (10, 50, 100, 500, and 1000 s^−1^) for 4% and 5% silica gel. To show how the viscosity of the intact gel changes as a function of time due to shear, Figure 6 illustrates the data obtained for 4% silica. The initial viscosity of the system is reduced over time when the shear rate is applied. There is a significant decay followed by a constant (equilibrium) value at long times. The data was used to calculate $a\left(\dot{\gamma }\right)$ using Equation (10) for different shear rates (see also Figure 1) and $c\left(\dot{\gamma }\right)$ from Equation (8). A power law dependence of $c\left(\dot{\gamma }\right)$ on the shear rate was observed with similar indexes describing the gels for both concentrations (Table 2). The constants $\lambda_{0}$ and $\lambda_{e}$ were also calculated from the tests performed by extrapolation of the viscosity curves at shear rate 0 and by the ratio between the experimentally measured value of $\eta_{\infty }$ in the capillary viscometer and the viscosity calculated by the flow curve of the broken fluid, respectively. Both constants also followed a power law dependence on the shear rate. A summary of the compiled data and constants obtained is presented in Table 2. For calculation of the power law relationships reported in Table 2, average values of the rheological parameters describing the Extended HB model were used. As illustrated in Figure 3, the rheological data exhibits a very small error.

**Figure 7:**
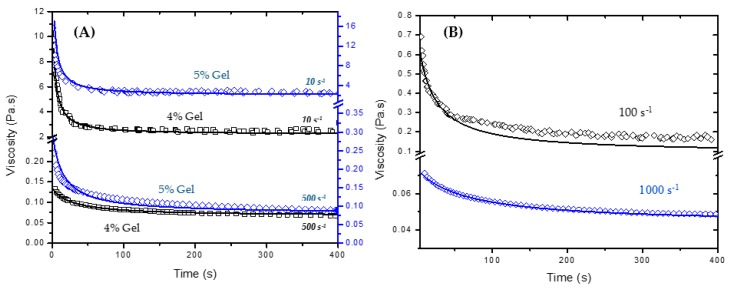
(**A**) Experimental (symbols) and predicted viscosity (lines) data for 4% and 5% gels at 10 and 500 s^−1^. The 4% data is plotted in the left *Y*-axis and is represented by square symbols. The 5% data is plotted in the right Y-axis and is represented by diamond symbols; (**B**) calculated viscosity assuming $\lambda_{e}=1$ for 4% gels at shear rates of 100 and 1000 s^−1^.

Experimental and fitted viscosity values for 4% and 5% silica gels are depicted in Figure 7A for two shear rates (10 and 500 s^−1^). The measured data shows good agreement with the fitted curve obtained by the approach developed in this work at high and low shear rates. For these curves, *λ_e_* was obtained from the viscosity measurements as a function of time for different shear rates. It is important to mention that for high shear rates, the equilibrium viscosity reaches values close to those provided by the flow curve of the broken gel (Extended HB Model), which implies that *λ_e_* is close to 1. In practice, the application of constant high shear rates for long times is also able to completely break down the 3D network formed by the colloidal particles. In this case, when *λ_e_* = 1, Equation (4) is simplified to:

(11) $$\lambda \left(\dot{\gamma },t\right)=\frac{\lambda_{0}+c\left(\dot{\gamma }\right)\cdot t\cdot \left(\lambda_{0}-1\right)}{1+c\left(\dot{\gamma }\right)\cdot t\cdot \left(\lambda_{0}-1\right)}$$

Taking $\lambda_{e}=1$ appears to be a good assumption for high shear rates; however, it is not as good for lower rates. Figure 7B shows the fitted and experimental viscosity values of 4% gels assuming $\lambda_{e}=1$ for two different shear rates (100 and 1000 s^−1^). The mechanical stresses applied to the system at high shear rates seems to be high enough to completely break down the gel network and to bring its viscosity down to those values obtained for the time independent system (see Table 1). The same cannot be said about lower shear rates. There is still a transient structure that affects the gel properties giving higher viscosity values to the fluid. These results show the important role of shear history for predicting the rheological properties of time dependent materials, especially colloidal gels. Moreover, they provide extensive experimental data that may help to elucidate the flow effects on the microstructure of time dependent colloidal gel systems.

## 5. Conclusions

The present article investigates the rheological behavior of time dependent colloidal gels made of hydrophilic fumed silica and a hydrophobic solvent. The results show how the viscosity of the gels is affected by the shear history. Findings suggest that the application of shear irreversibly changes the microstructure of the colloidal gels, their 3D network, and consequently their flow properties. Shear forces affect not only the viscosity of the gel system, but also its elasticity (changes in normal stresses). These effects result in structural changes in the material with the breakdown of the gel percolated network and inter-aggregate links present in the gel system. Cryo-SEM shows different patterns for the intact and sheared gels, which may be an indication of changes in the organizational structure of the system caused by shear. After the application of high shear for a relatively long time, time dependence in the gels is no longer observed and the shear history becomes irrelevant. To model the structural changes during flow, the Tiu and Boger model was used with modifications to consider the flow curve of the pre-sheared material as the constitutive model. Good agreement between experimental and predicted data was observed for these colloidal gels in a relatively wide range of shear rates. Therefore, this approach provides a more realistic method to analyze the rheological properties of time dependent systems than the currently used Tiu and Boger model.

## Figures and Tables

**Table 1:** Extended Herschel-Bulkley parameters (Equation (2)) for sheared colloidal gels.

Herschel-Bulkley Parameters	4.0%	5.0%	6.0%	7.0%
$\tau_{0}$ (Pa)	0.6 ± 0.07	2.4 ± 0.2	5.5 ± 0.4	5.0 ± 0.3
*K* (Pa·s^n^)	0.24 ± 0.03	0.58 ± 0.04	0.98 ± 0.1	1.67 ± 0.1
*n*	0.71	0.57	0.45	0.29
*η*_∞_ (Pa·s)	0.01	0.01	0.01	0.01
*R*^2^	0.97	0.99	0.99	0.99

* n is the power law index.

**Table 2:** Structural kinetic parameters to describe viscosity time decay for 4% and 5% gels.

Structural Kinetic Parameters	4.0%	*R*^2^	5.0%	*R*^2^
$c\left(\dot{\gamma }\right)$	$0.0028{\dot{\gamma }}^{0.36}$	0.99	$0.0026{\dot{\gamma }}^{0.33}$	0.98
$\lambda_{e}$	$27.6{\dot{\gamma }}^{-0.48}$	0.98	$7.17\;{\dot{\gamma }}^{-0.26}$	0.96
$\lambda_{0}$	$582.6{\dot{\gamma }}^{-0.85}$	0.99	$4.1{\dot{\gamma }}^{-0.68}$	0.98

## References

[1] Barnes H.A. (1997). Thixotropy a review. J. Non-Newton. Fluid.

[2] Mewis J., Wagner N.J. (2009). Thixotropy. Adv. Colloid Interface Sci..

[3] Mokoonlall A., Nöbel S., Hinrichs J. (2016). Post-processing of fermented milk to stirred products: Reviewing the effects on gel structure. Trends Food Sci. Technol..

[4] Bazmi A., Relkin P. (2009). Effects of processing conditions on structural and functional parameters of whipped dairy emulsions containing various fatty acid compositions. J. Dairy Sci..

[5] Barbucci R., Pasqui D., Favaloro R., Panariello G. (2008). A thixotropic hydrogel from chemically cross-linked guar gum: Synthesis, characterization and rheological behaviour. Carbohydr. Res..

[6] Lee C.H., Moturi V., Lee Y. (2009). Thixotropic property in pharmaceutical formulations. J. Control. Release.

[7] Labanda J., Llorens J. (2008). Effect of aging time on the rheology of Laponite dispersions. Colloid Surf. A Physicochem. Eng. Asp..

[8] Bosma M., Brinkhuis R., Coopmans J., Reuvers B. (2006). The role of sag control agents in optimizing the sag/leveling balance and a new powerful tool to study this. Prog. Org. Coat..

[9] Phuoc T.X., Howard B.H., Chyu M.K. (2009). Synthesis and rheological properties of cation-exchanged Laponite suspensions. Colloid Surf. A Physicochem. Eng. Asp..

[10] Labanda J., Llorens J. (2005). Influence of sodium polyacrylate on the rheology of aqueous Laponite dispersions. J. Colloid Interface Sci..

[11] Nosrati A., Addai-Mensah J., Skinner W. (2011). Rheology of aging aqueous muscovite clay dispersions. Chem. Eng. Sci..

[12] Pek Y.S., Wan A.C., Shekaran A., Zhuo L., Ying J.Y. (2008). A thixotropic nanocomposite gel for three-dimensional cell culture. Nat. Nanotechnol..

[13] Arnold R., Santos P.H.S., Campanella O.H., Anderson W.E. (2011). Rheological and thermal behavior of gelled hydrocarbon fuels. J. Propuls. Power.

[14] Rahimi S., Natan B. (2000). Thixotropic effect of inorganic gel fuels. J. Propuls. Power.

[15] Jyoti B.V., Baek S.W. (2016). Rheological characterization of ethanolamine gel propellants. J. Energ. Mater..

[16] Steffe J.F. (1996). Rheological Methods in Food Process Engineering.

[17] Rajaram B., Mohraz A. (2010). Microstructural response of dilute colloidal gels to nonlinear shear deformation. Soft Matter.

[18] Labanda J., Marco P., Llorens J. (2004). Rheological model to predict the thixotropic behaviour of colloidal dispersions. Colloid Surface A Physicochem. Eng. Asp..

[19] Labanda J., Llorens J. (2006). A structural model for thixotropy of colloidal dispersions. Rheol. Acta.

[20] Santos P.H.S., Campanella O.H., Carignano M.A. (2013). Effective attractive range and viscoelasticity of colloidal gels. Soft Matter.

[21] Santos P.H.S., Campanella O.H., Carignano M.A. (2010). Brownian dynamics study of gel-forming colloidal particles. J. Phys. Chem. B.

[22] Lu P.J., Zaccarelli E., Ciulla F., Schofield A.B., Sciortino F., Weitz D.A. (2008). Gelation of particles with short-range attraction. Nature.

[23] Zaccarelli E. (2007). Colloidal gels: Equilibrium and non-equilibrium routes. J. Phys. Condens. Matter.

[24] (2009). CAB-O-SIL Untreated Fumed Silica: Propertiesand Functions, Mechanisms of CAB-O-SIL, Technical Bulletin. http://www.cabotcorp.com/.

[25] Dullaert K., Mewis J. (2005). Thixotropy: Build-up and breakdown curves during flow. J. Rheol..

[26] Dullaert K., Mewis J. (2005). A model system for thixotropy studies. Rheol. Acta.

[27] Willenbacher N. (1996). Unusual thixotropic properties of aqueous dispersions of Laponite RD. J. Colloid Interface Sci..

[28] Rueb C.J., Zukoski C.F. (1997). Viscoelastic properties of colloidal gels. J. Rheol..

[29] Tiu C., Boger D.V. (1974). Complete rheological characterization of time-dependent food products. J. Texture Stud..

[30] Denn M.M., Bonn D. (2011). Issues in the flow of yield-stress liquids. Rheol. Acta.

[31] Arnold R., Santos P.H., Kubal T., Campanella O., Anderson W.E. Investigation of gelled JP-8 and RP-1 fuels. Proceedings of the World Congress on Engineering and Computer Science.

